# Predicting level 2 axillary lymph node metastasis in a Chinese breast cancer population post-neoadjuvant chemotherapy: development and assessment of a new predictive nomogram

**DOI:** 10.18632/oncotarget.16131

**Published:** 2017-03-15

**Authors:** Caigang Liu, Yanlin Jiang, Xin Gu, Zhen Xu, Liping Ai, Hao Zhang, Guanglei Chen, Lisha Sun, Yue Li, Hong Xu, Huizi Gu, Ying Yu, Yangyang Xu, Qiyong Guo

**Affiliations:** ^1^ Department of Breast Surgery, Shengjing Hospital of China Medical University, Shenyang, China; ^2^ Department of Breast Disease and Reconstruction Center, Breast Cancer Key Lab of Dalian, the Second Hospital of Dalian Medical University, Dalian, China; ^3^ Department of Head and Neck Surgery, Harbin Medical University Cancer Hospital, Harbin, China; ^4^ Department of Surgical Oncology, the First Hospital of China Medical University, Shenyang, China; ^5^ Department of Breast Surgery, Liaoning Cancer Hospital & Institute, Shenyang, China; ^6^ Department of Internal Neurology, the Second Hospital of Dalian Medical University, Dalian, China; ^7^ Liaoning Medical Device Test Institute, Shenyang, China; ^8^ Department of Urinary Surgery, Harbin Medical University Cancer Hospital, Harbin, China; ^9^ Department of Radiology, Shengjing Hospital of China Medical University, Shenyang, China

**Keywords:** breast cancer, neoadjuvant chemotherapy, level 2 axillary lymph node metastasis, nomogram, level 2 axillary lymph node dissection

## Abstract

**Background:**

We aimed to develop a new nomogram to predict the probability of level 2 axillary lymph node metastasis (L-2-ALNM) in breast cancer (BC) patients treated with neoadjuvant chemotherapy (NAC).

**Methods:**

Data were collected from 709 patients who received neoadjuvant chemotherapy and then underwent axillary lymph node (ALN) dissection between May 2009 and December 2015 at the Liaoning Cancer Hospital. The level 2 axillary lymph node metastasis (L-2-ALNM ) nomogram was created from the logistic regression model. An additional set of 141 consecutive patients treated at the same institution between January 2015 and December 2015 were enrolled as the validation group. The predictive accuracy of the L-2-ALNM nomogram was measured by calculating the area under the receiver operating characteristic curve (AUC).

**Results:**

In multivariate analysis, age, tumor size, histological grade, skin invasion, and response to neoadjuvant chemotherapy were identified as independent predictors of L-2-ALNM. The new model was accurate and discriminating for both the modeling and validation groups (AUC: 0.819 *vs* 0.849). The false-negative rates of the L-2-ALNM nomogram were 4.44% and 7.69% for the predicted probability cut-off points of 10% and 20%.

**Conclusion:**

The L-2-ALNM nomogram shows reasonable accuracy for making clinical decisions. The omission of level 2 axillary lymph node dissection after neoadjuvant chemotherapy might be possible if the probability of level 2 lymph node involvement was < 10% or < 20% in accordance with the acceptable risk determined by medical staff and patients.

## BACKGROUND

According to reports, BC has become one of the most common malignancies in American women, with its incidence continuously rising. Neoadjuvant therapy can lower tumor burden and increase breast conservation rate. At the same time, response to chemotherapy can be evaluated, hence this therapy is widely used [1]. In this era of individually and precisely tailored treatments, patients without L-2-ALNM after NAC may be safely treated without level 2 axillary lymph node dissection (L-2-ALND). To identify patients in whom L-2-ALND may be omitted, a noninvasive method approximating the accuracy of L-2-ALND is needed to evaluate the level 2 axillary lymph node response to NAC.

In China, if patients undergo neoadjuvant chemotherapy, the preferred surgical approach includes ALN dissection (ALND) (both level 1 and level 2 ALNs are dissected) in addition to surgical treatment of the primary tumor, regardless of the level 2 axillary node response to NAC. ALND is unlikely to improve locoregional control and survival in patients without level 2 axillary lymph node metastasis, although this has never been confirmed in a randomized controlled trial. Identifying patients with uninvolved level 2 axillary lymph nodes and subsequent omission of L-2-ALND could prevent the short and long term side-effects of this procedure such as lymph edema, limited range of motion of the shoulder, and numbness of the upper arm [2-5].

ALND has been the gold standard to ascertain the distribution of ALN involvement for breast cancer patients receiving NAC. However, there are no noninvasive techniques currently available that approximate the accuracy of ALND for identifying L-2-ALNM in breast cancer patients post NAC. The purpose of this study is to establish a predictive model that approximates the accuracy of ALND for identifying patients without L-2-ALNM after NAC.

## MATERIALS AND METHODS

### Patients

Data were collected from 709 patients with breast cancer treated with NAC between 2009 and 2015 at the Liaoning Cancer Hospital. The eligibility criteria were: (1) finished different cycles of planned-dose neoadjuvant chemotherapy, (2) underwent both radical excision of the primary tumor and ALND, (3) complete information on preoperative immunohistochemistry (IHC) (including estrogen receptor [ER], progesterone receptor [PrR], HER-2, and Ki67) was available, and (4) tumor type was invasive ductal carcinoma. Patients with missing data, or with distant metastatic disease, or who underwent radiotherapy preoperatively were excluded.

The patients were divided into a modeling group (nomogram construction) and a validation group (nomogram validation). The modeling group included 568 breast cancer patients who underwent ALND after neoadjuvant chemotherapy between May 2009 and December 2014. The validation group included 141 breast cancer patients who underwent ALND between January 2015 and December 2015. The protocol was approved by the Ethics Committee of LiaoNing Cancer Hospital. All patients gave informed consent prior to inclusion in the study.

### Treatment

The patients in our cohort received NAC for a median of 4 cycles (range, 2-6 cycles). Surgical treatment was performed in accordance with the Chinese breast cancer guidelines. Surgery consisted of resection of the primary tumor (either by mastectomy or by wide local excision followed by radiation therapy) and performance of level 1 and level 2 ALND. The quantity and histological status of the level 1and level 2 nodes were recorded.

### Data extraction

Data were extracted from patient records regarding age at diagnosis, menopausal status, tumor characteristics (tumor location; histological type; histological grade; skin invasion; preoperative and postoperative clinical tumor size based on a combination of imaging and physical examination; histologic subtype, the status of ER, PrR, and HER2, and the Ki-67 index, based on core biopsy results obtained preoperatively), cycles of planned-dose neoadjuvant chemotherapy, and postoperative pathologic characteristics of axillary lymph nodes including level 1 and level 2. Response to neoadjuvant chemotherapy was in accordance with RECIST evaluation criteria except that a complete remission (CR) only refers to the breast tumor rather than the axillary node lymph nodes.

### Pathologic evaluation

Core biopsy samples and surgical specimens were evaluated according to the Chinese breast cancer guidelines. The initial core biopsy sample of the primary tumor was evaluated using standard hematoxylin and eosin staining, IHC, and fluorescence or chromogenic in situ hybridization (FISH) (or both) to determine the histological subtype, the Ki67 index, and the status of ER, PrR, and HER2. Cut-off values for ER, PR, and Ki67 were 10%, 10%, and 20% [6], respectively. The cut-off value for HER2 scoring was set at 3+ for IHC and 2.0 for FISH. Any cases of HER2 found to be 2+ on IHC were examined by FISH and classified as HER2-positive if the *HER2* gene was found to be amplified.

### Statistical analysis

The chi-square test or Fisher’s exact test was utilized for categorical data, whereas descriptive statistics and t-tests were used for the between-group or within-group comparisons of independent samples. For development of the prediction model, predefined variables were included in a multivariate binary logistic regression analysis to determine corresponding regression coefficients with 95% confidence intervals (CIs). The predefined variables were age at diagnosis, menopausal status, clinical tumor size, tumor location, cycles of planned-dose neoadjuvant chemotherapy, histological grade, ER, PrR, HER2, Ki-67, skin invasion, and response to neoadjuvant chemotherapy. These variables were included in a binary logistic regression analysis using a forward selection procedure in order to identify the independent risk factors for the Level 2 Axillary Lymph Node Metastasis nomogram. Variables with a p-value less than 0.05 in the multivariate analysis, as independent risk factors, were included in an equation for predicting the probability of L-2-ALNM. Coefficients for each variable and the constant in the equation were generated based on multivariate analysis. A nomogram was developed to be a graphic representation of the model. The predictive model was then validated with an additional set of 141 Chinese patients in the validation group. The receiver operating characteristic (ROC) curve was drawn, and the area under the curve was used to assess the predictive accuracy of the model. As a general rule, a model that performs with an AUC curve of 0.7-0.8 is considered acceptable, and an AUC of 0.81-0.9 indicates the model shows excellent discrimination. A calibration plot with bootstrapping was also used to illustrate the association between the actual probability and the predicted probability [7]. The goodness of fit of the model was assessed by the Hosmer and Lemeshow test, and p > 0.05 indicated a good fit [8,9]. All reported p values are two-sided. Statistical analysis was performed by using the statistical software package SPSS (version 17, SPSS Inc., Chicago, IL, USA) and R software (version 3.1.0).

## RESULTS

This study included data from 709 patients, with 568 patients in the modeling group and 141 patients in the validation group. The clinicopathological characteristics of both groups are summarized in Table 1. Among the 709 patients, 188 were pathologically confirmed to have L-2-ALNM postoperatively. There was no statistically significant difference between the modeling group and the validation group except for tumor location and PrR status. In the multivariate analysis, several independent predictors of L-2-ALNM post NAC were identified (Table 2) including age at diagnosis (p = 0.007), clinical tumor size (p < 0.001), skin invasion (p < 0.001), histological grade (p = 0.005), and response to NAC (p < 0.001).

**Table 1:** Comparison of descriptive characteristics of the modeling and validation groups for the L-2-ALNM nomogram.

Variables	Modeling No. (%)	Validation No. (%)	*P*-value
No. of Patients	568 (100%)	141 (100%)	
L-2-ALNM			0.731
Yes	149 (26.2)	39 (27.7)	
No	419 (73.8)	102 (72.3)	
Age (year)			0.764
≤45	148（80.9）	420（79.8）	
>45	35（18.1）	106（20.2）	
Menopausal status			0.090
Premenopausal	319（56.2）	68（48.2）	
Postmenopausal	249（43.8）	73（51.8）	
Tumor size (cm) Median (range)	3.5 (1.6-9.8)	3.5 (1.7-11.0)	0.688
Histological grade			0.255
Ⅰ	17 （3.0）	1（0.7）	
Ⅱ	491（86.4）	127 (90.7）	
Ⅲ	60 （10.6）	12（8.6）	
ER status			0.741
Positive	363（63.9）	88（62.4）	
Negative	205（36.1）	53（37.6）	
PrR status			0.001
Positive	309（54.4）	54 (38.3)	
Negative	259（45.6）	87（61.7）	
Her-2 status			0.105
Positive	110（19.4）	19（13.5）	
Negative	458（80.6）	122（86.5）	
Ki-67 status			0.466
≤20	393 (69.2)	102 (72.3)	
＞20	175 (30.8)	39 (27.7)	
(ER/PrR) status			0.570
Positive	373 (65.7)	89(63.1)	
Negative	195 (34.3)	52(36.9)	
Skin invasion			0.888
Yes	74 (13.0)	19 (13.5)	
No	494 (87.0)	122 (86.5)	
Rresponse to NAC			0.260
PD	12 （2.1）	4 （2.8）	
SD	173（30.5）	33（23.4）	
PR	340（59.9）	89（63.1）	
CR	43 （7.6）	141（10.6）	
Cycles of NAC Median (range)	4 (2-6)	4 (2-6)	0.053
Tumor location			0.029
UOQ	364 (64.1)	76 (53.9)	
LOQ	34 (6.0)	10 (7.1)	
LIQ	18 (3.2)	6 (4.3)	
UIQ	108 (18.5)	25 (17.7)	
Central	47 (8.3)	24 (17.0)	

Comparison between the modeling group and validation group by clinicopathological characteristics. Abbreviations:UOQ, upper outer quadrant; UIQ, upper inner quadrant; LOQ, lower outer quadrant; LIQ, lower inner quadrant; L-2-ALNM, level 2 axillary lymph node metastasis; NAC, neoadjuvant chemotherapy; PD, progressive disease; SD, stable disease; PR, partial remission; CR, complete remission.

**Table 2:** Results of multivariate logistic regression testing the association of each variable with L-2-ALNM.

Variables	Coefficient	SE.	Wald value	P value	OR	95 % CI	
						Lower	Upper
Age	-0.680	0.252	7.310	0.007	0.506	0.309	0.829
Histological grade	0.835	0.296	7.958	0.005	2.305	1.290	4.116
Tumor size	0.579	0.084	47.123	<0.001	1.785	1.513	2.105
Rresponse to NAC	-1.475	0.203	52.651	<0.001	0.229	0.154	0.341
Skin invasion	1.163	0.309	14.172	<0.001	3.199	1.746	5.862
Constant	-0.906	0.848	1.141	0.285	0.404		

In the modeling group, 259 patients (45.6%) were PrR positive, in contrast to only 87 patients (61.7%) in the validation group. However, the rates of ER/PrR positivity in both groups were comparable, and corresponded to 65.7% in the modeling group and 63.1% in the validation group. As to the tumor position, besides the central location and upper outer quadrants, there were no obvious differences between the remaining groups. Tumors in the central location made up to 8.3% and 17.1% of all tumors in the modeling and validation groups, respectively. In contrast, there is a smaller percentage of upper outer quadrant in the validation cohort (53.9%) compared to the modeling cohort (64.1%).We believe that the difference might be a random fluctuation due to the relatively small numbers of patients.

**Figure 1:**
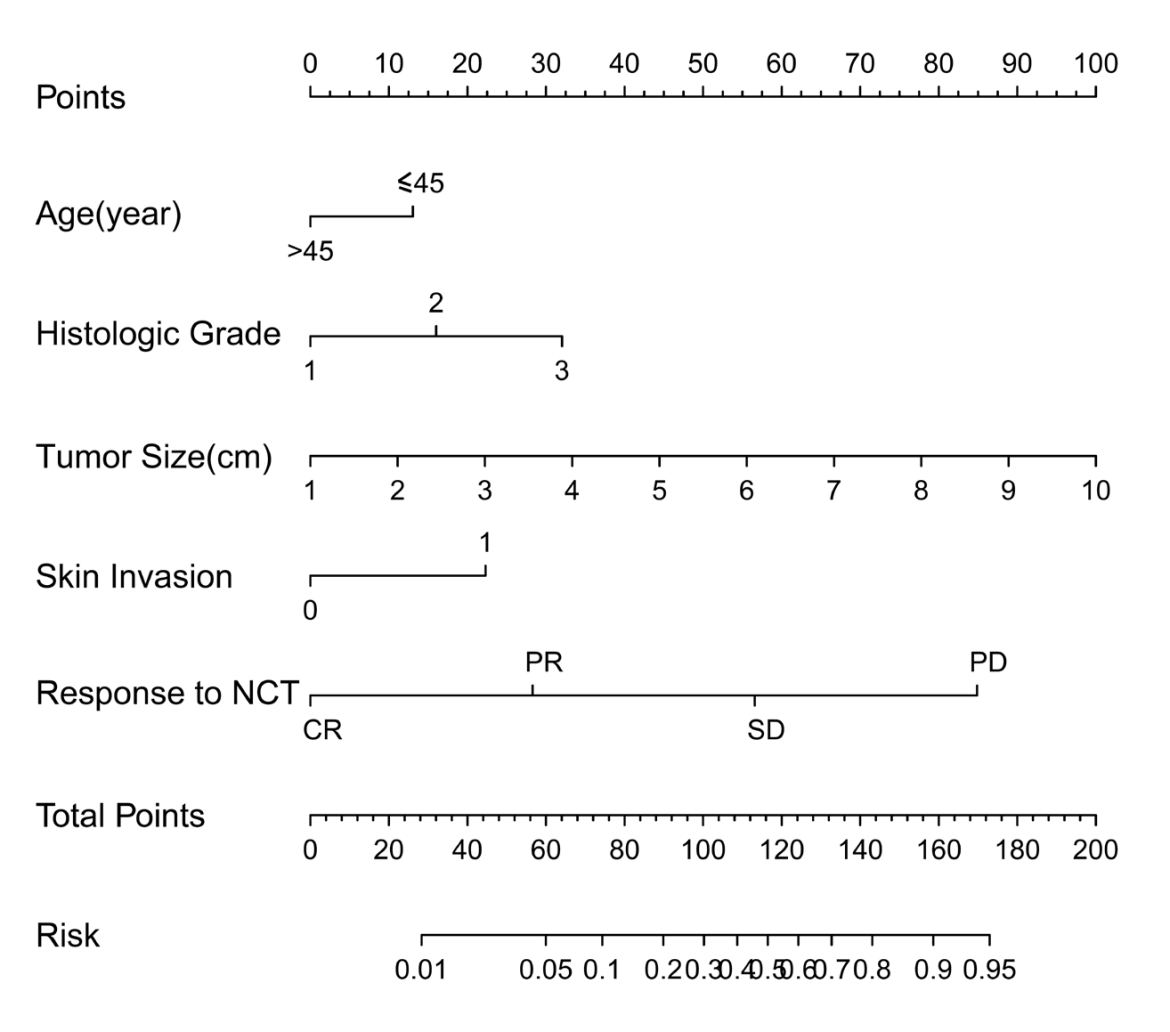
Nomogram for predicting the probability of L-2-ALNM The nomogram consists of eight rows. The first row is the point assignment for each variable. For an individual patient, each variable is assigned a point value according to the clinicopathological characteristics by drawing a vertical line between the exact variable value and the points line. Subsequently, a total point score (row 7) can be obtained by summing all of the assigned points for the five variables. Finally, the predictive probability of axillary metastasis can be obtained by drawing a vertical line between the total points and risk (the final row).

**Figure 2:**
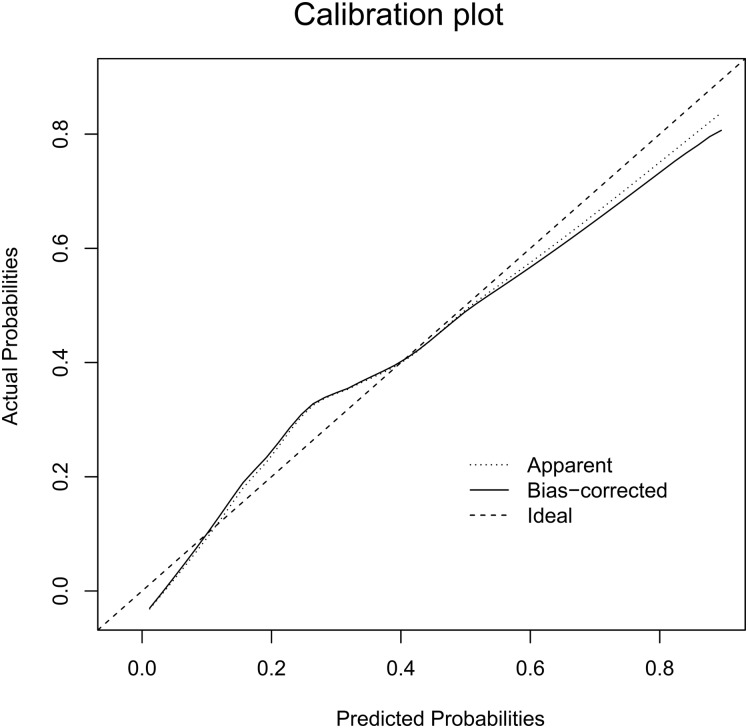
Calibration plot of the nomogram for the probability of L-2-ALNM (bootstrap 1000 repetitions) The reference line represents perfect equality of the predicted probability and the actual incidence of L-2-ALNM.

### Development of the L-2-ALNM nomogram

The L-2-ALNM risk was expressed by the following equation: ln (p/1 − p) = 0.579 × a − 0.68 × b + 0.835 × c − 1.475 × d + 1.163 × e -0.90.

The equation is explained as follows: p = the probability of L-2-ALNM; a = tumor size in cm; b = age (0 if age ≤ 45 years, 1 if age > 45 years); c = histological grade (1 if grade 1, 2 if grade 2, and 3 if grade 3); d = response to neoadjuvant chemotherapy (1 if progressive disease [PD], 2 if stable disease [SD], 3 if partial remission [PR], and 4 if complete remission [CR]); and e = skin invasion (0 if no, 1 if yes).

**Figure 3:**
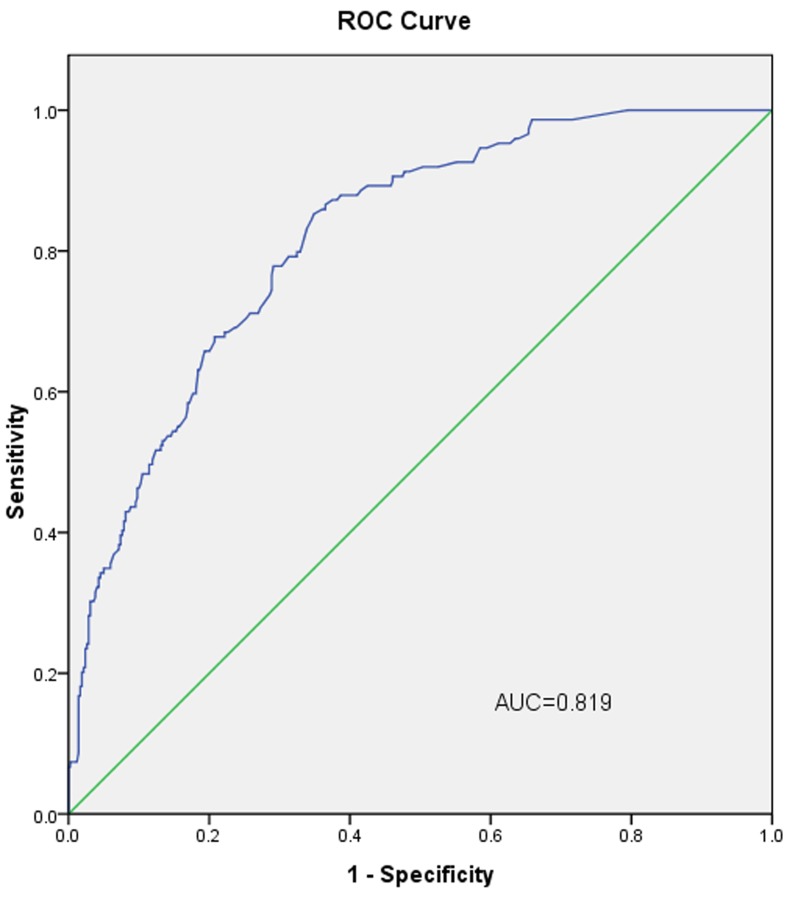
The ROC curve calculation for the L-2-ALNM nomogram applied to the modeling group (*n* = 568) The AUC is 0.819, 95% confidence interval (95% CI, 0.783 to 0.856).

The L-2-ALNM nomogram (Figure 1) is based on the results of the logistic regression analysis (Table 2) in the modeling group, and is composed of the following variables: (1) age at diagnosis, (2) clinical tumor size, (3) histological grade, (4) skin invasion, and (5) response to NAC. In the modeling group, the AUC was 0.819 (Figure 3).

### Validation of the L-2-ALNM nomogram

**Figure 4:**
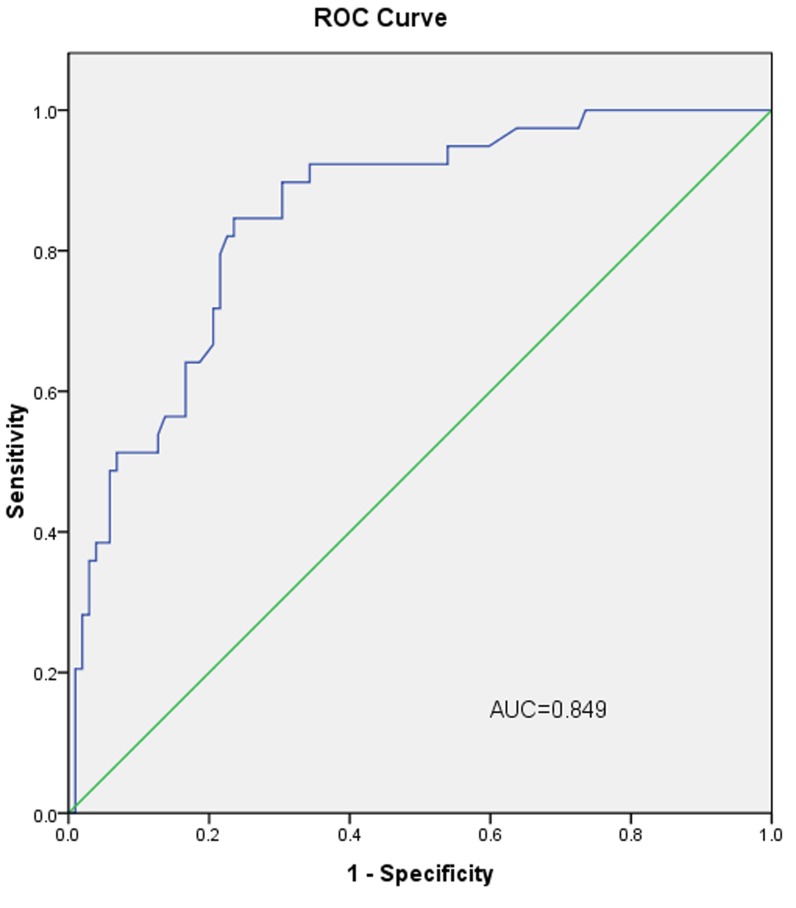
The ROC curve calculation for the L-2-ALNM nomogram applied to the validation group (*n* = 141) The AUC is 0.849 (95% CI, 0.782 to 0.917).

In the validation group, the AUC of L-2-ALNM nomogram was 0.849 (Figure 4). When the cut-off value was 10%, the false negative rate of the prediction model was only 4.44%. The subgroup with low risk of L-2-ALNM accounted for 31.91% (45/141) of all the population (Table 3).

**Table 3:** Predictive values of the L-2-ALNM nomogram at different cutoff values in the validation cohort.

Cut-off values(%)	No. of patients and percentage(%)	False-negative rate (%)	No. of falsenegative patients	Negative predictive value (%)
<10	45(31.91)	4.44	2	95.56
<15	66(46.84)	4.55	3	95.45
<20	78(55.32)	7.69	6	92.31
<30	94(66.67)	13.83	13	86.17

The Hosmer-Lemeshow goodness-of-fit test (p = 0.25) indicated that our nomogram fits well. As seen in Figure 2, the bias-corrected curve and the ideal curve were very close, indicating a well-calibrated nomogram.

## DISCUSSION

As knowledge regarding disease heterogeneity increases, breast cancer therapy has entered into an age of personalized and precision therapy. However, there is still controversy regarding whether or not L-2-ALND should be carried out in patients under NAC. Indeed, previous studies have shown that the incidence of post-operative upper extremity lymphedema, upper limb numbness, limited shoulder joint mobility, and other side effects in patients with reduced lymph node clearance was significantly reduced compared with patients with complete axillary lymph node clearance. Therefore, it is important to determine accurately if patients have L-2-ALNM, in order to be able to exempt some patients from level 2 axillary lymph node clearance.

Currently, the modified radical mastectomy is still one of the commonly used local treatments in patients who have undergone neoadjuvant therapy in China. To date, there have been no reports on predictive models on L-2-ALNM following neoadjuvant chemotherapy. Even though challenging, developing an accurate and non-invasive model for preoperative evaluation of L-2-ALNM before surgery is extremely important for the optimization of the treatment protocol. Therefore, this study has developed a predictive model to evaluate the probability of L-2-ALNM after neoadjuvant therapy and before surgery.

This model can identify the patients with breast cancer who do not have L-2-ALNM after NAC, thus sparing them highly invasive procedures, such as the L-2-ALND. The model offers several advantages compared with alternative methods for assessing axillary metastasis after neoadjuvant chemotherapy. First, the omission of L-2-ALND for a lesser invasive surgical procedure, which may decrease adverse events, such as lymph edema. Second, there is the possible cost reduction related to eliminating the need for additional surgical procedures, pathologic examination of the lymph nodes, and diagnostic tests.

The patient and tumor characteristics of 709 patients who underwent NAC and subsequent ALND were analyzed retrospectively. The predictive value curve and actual value curves showed similar trends, with no obvious deviations. In the validation group, the AUC value was 0.849, demonstrating a better discrimination ability. However, some clinicopathological variables showed statistical differences between the modeling and validation groups, which could be due to the small sample size used for comparison. The age at diagnosis, clinical tumor size, histological grade, skin invasion, and response to neoadjuvant chemotherapy were matched closely between the modeling and validation groups, which justifies why the two groups performed equally well, despite them bearing significant differences in tumor location and PrR status.

Several previous studies have verified the variables associated with axillary lymph node metastases. According to the multivariate analysis, our predictive model consisted of five variables that are associated with L-2-ALNM. These were age, histological grade, tumor size, skin invasion, and response to neoadjuvant chemotherapy. Indeed, tumor size, histological grade, and skin invasion all have been previously proven to be a risk factor of axillary lymph node metastasis [5,10-15], which is consistent with our results. Previous studies have shown high concordance rates of age and response to NAC between primary and axillary lymph node metastatic lesions [16-21].

To our knowledge, the L-2-ALNM nomogram was first reported in the English literature. Our L-2-ALNM nomogram showed a better predictive power in both the validation and modeling groups (AUC = 0.819 and 0.849, respectively). Generally, a model with a good discrimination ability is represented by an AUC value ranging between 0.81 and 0.9. In our model, these five variables that were associated with L-2-ALNM were readily available prior to surgery, thus increasing the practicality of our model. The results of our study suggest that this nomogram is reliable and useful in Chinese populations.

When this model was applied in the validation group, we calculated the false **negativity** rates at cut-off values of 10%, 15%, 20%, and 30%, to further evaluate the model’s clinical practicality. During these calculations, we emphasized that only populations with low risk of metastases were exempted from L-2-ALND.

According to reports by the American Society of Clinical Oncology expert groups [22, 23], the false negativity rate of sentinel lymph node biopsy is 8.4% (range 0-29%). Therefore, we hypothesized that for a predictive nomogram, most surgeons could accept a false negativity rate of 0-8%. Hence, when the cut-off values were set to 10% and 20%, the false negativity rates were 4.44% and 7.69%, respectively, showing that our nomogram is acceptable. At the predictive probability cut-off value of 10%, the false negativity rate of the nomogram was only 4.44%. This subgroup with low probability of lymph node metastasis made up about 31.91% (45/141) of the patients in the validating group. In these 45 patients, only two had L-2-ALNM, hence the negative predictive value was 95.56%. When the false negativity rate was increased to 7.69%, patients in the low risk subgroup made up about 55.32% of the patients. Therefore, the nomogram demonstrated a better practicality in these low risk subgroups. Hence, when the L-2-ALNM rate is < 10% or < 20%, the patient could be exempted from L-2-ALND.

Treatment of axillary lymph nodes in patients with breast cancer has been continuously evolving. The treatment protocols for axillary lymph nodes are gradually increasing. Indeed, recent clinical trial results have shown that predicting whether there are metastases in the remaining lymph nodes in patients with breast cancer is becoming increasingly important. The accuracy of predicting L-2-ALNM after neoadjuvant chemotherapy can be improved by using our nomogram. Our model provides a novel, reliable, convenient, and safe method for processing axillary lymph nodes in patients who have had neoadjuvant chemotherapy.

Nevertheless, several limitations are worth noting in our present study. First, our observations are limited to retrospective study from a single center. Second, only the invasive ductal carcinoma was included as the tumor type, while patients who received radiotherapy preoperatively were excluded, which decreases the scope of the model’s application. Moreover, the development of prediction models has innate limitations. Therefore, there is a need for prospective studies from larger samples to verify this type of predictive model [24].

## CONCLUSIONS

We developed a nomogram used to be predict post-neoadjuvant chemotherapy L-2-ALNM. With this nomogram, we can accurately predict post-neoadjuvant chemotherapy level 2 lymph node metastasis and avoid unnecessary level 2 axillary lymph node dissection.

## References

[1] Generali D, Ardine M, Strina C, Milani M, Cappelletti MR, Zanotti L, Forti M, Bedussi F, Martinotti M, Amoroso V, Sigala S, Simoncini E, Berruti A, Bottini A (2015). Neoadjuvant Treatment Approach: The Rosetta Stone for Breast Cancer?. Journal of the National Cancer Institute Monographs.

[2] Jung SY, Shin KH, Kim M, Chung SH, Lee S, Kang HS, Lee ES, Kwon Y, Lee KS, Park IH, Ro J (2014). Treatment factors affecting breast cancer-related lymphedema after systemic chemotherapy and radiotherapy in stage II/III breast cancer patients. Breast cancer research and treatment.

[3] Ashikaga T, Krag DN, Land SR, Julian TB, Anderson SJ, Brown AM, Skelly JM, Harlow SP, Weaver DL, Mamounas EP, Costantino JP, Wolmark N (2010). Morbidity results from the NSABP B-32 trial comparing sentinel lymph node dissection versus axillary dissection. Journal of surgical oncology.

[4] Liu CQ, Guo Y, Shi JY, Sheng Y (2009). Late morbidity associated with a tumour-negative sentinel lymph node biopsy in primary breast cancer patients: a systematic review. European journal of cancer.

[5] Schipper RJ, Moossdorff M, Nelemans PJ, Nieuwenhuijzen GA, de Vries B, Strobbe LJ, Roumen RM, van den Berkmortel F, Tjan-Heijnen VC, Beets-Tan RG, Lobbes MB, Smidt ML (2014). A model to predict pathologic complete response of axillary lymph nodes to neoadjuvant chemo(immuno)therapy in patients with clinically node-positive breast cancer. Clinical breast cancer.

[6] Coates AS, Winer EP, Goldhirsch A, Gelber RD, Gnant M, Piccart-Gebhart M, Thurlimann B, Senn HJ (2015). Tailoring therapies--improving the management of early breast cancer: St Gallen International Expert Consensus on the Primary Therapy of Early Breast Cancer 2015. Annals of oncology.

[7] Harrell FE, Lee KL, Mark DB (1996). Multivariable prognostic models: issues in developing models, evaluating assumptions and adequacy, and measuring and reducing errors. Statistics in medicine.

[8] Hosmer DW, Lemeshow S

[9] Jin X, Jiang YZ, Chen S, Shao ZM, Di GH (2016). A Nomogram for Predicting the Pathological Response of Axillary Lymph Node Metastasis in Breast Cancer Patients. Scientific reports.

[10] Xie F, Yang H, Wang S, Zhou B, Tong F, Yang D, Zhang J (2012). A logistic regression model for predicting axillary lymph node metastases in early breast carcinoma patients. Sensors (Basel, Switzerland).

[11] Bevilacqua JL, Kattan MW, Fey JV, Cody HS, Borgen PI, Van Zee KJ (2007). Doctor, what are my chances of having a positive sentinel node? A validated nomogram for risk estimation. Journal of clinical oncology.

[12] Meretoja TJ, Heikkila PS, Mansfield AS, Cserni G, Ambrozay E, Boross G, Zgajnar J, Perhavec A, Gazic B, Arisio R, Tvedskov TF, Jensen MB, Leidenius MH (2014). A predictive tool to estimate the risk of axillary metastases in breast cancer patients with negative axillary ultrasound. Annals of surgical oncology.

[13] Greer LT, Rosman M, Charles Mylander W, Liang W, Buras RR, Chagpar AB, Edwards MJ, Tafra L (2014). A prediction model for the presence of axillary lymph node involvement in women with invasive breast cancer: a focus on older women. The breast journal.

[14] Viale G, Zurrida S, Maiorano E, Mazzarol G, Pruneri G, Paganelli G, Maisonneuve P, Veronesi U (2005). Predicting the status of axillary sentinel lymph nodes in 4351 patients with invasive breast carcinoma treated in a single institution. Cancer.

[15] Klar M, Foeldi M, Markert S, Gitsch G, Stickeler E, Watermann D (2009). Good prediction of the likelihood for sentinel lymph node metastasis by using the MSKCC nomogram in a German breast cancer population. Annals of surgical oncology.

[16] Wasuthit Y, Kongdan Y, Suvikapakornkul R, Lertsithichai P, Chirappapha P (2011). Predictive factors of axillary lymph node metastasis in breast cancer. Journal of the Medical Association of Thailand.

[17] Alvarado R, Yi M, Le-Petross H, Gilcrease M, Mittendorf EA, Bedrosian I, Hwang RF, Caudle AS, Babiera GV, Akins JS, Kuerer HM, Hunt KK (2012). The role for sentinel lymph node dissection after neoadjuvant chemotherapy in patients who present with node-positive breast cancer. Annals of surgical oncology.

[18] Koolen BB, Valdes Olmos RA, Wesseling J, Vogel WV, Vincent AD, Gilhuijs KG, Rodenhuis S, Rutgers EJ, Vrancken Peeters MJ (2013). Early assessment of axillary response with (1)(8)F-FDG PET/CT during neoadjuvant chemotherapy in stage II-III breast cancer: implications for surgical management of the axilla. Annals of surgical oncology.

[19] Rouzier R, Extra JM, Klijanienko J, Falcou MC, Asselain B, Vincent-Salomon A, Vielh P, Bourstyn E (2002). Incidence and prognostic significance of complete axillary downstaging after primary chemotherapy in breast cancer patients with T1 to T3 tumors and cytologically proven axillary metastatic lymph nodes. Journal of clinical oncology.

[20] Straver ME, Rutgers EJ, Russell NS, Oldenburg HS, Rodenhuis S, Wesseling J, Vincent A, Peeters MT (2009). Towards rational axillary treatment in relation to neoadjuvant therapy in breast cancer. European journal of cancer.

[21] von Minckwitz G, Untch M, Blohmer JU, Costa SD, Eidtmann H, Fasching PA, Gerber B, Eiermann W, Hilfrich J, Huober J, Jackisch C, Kaufmann M, Konecny GE (2012). Definition and impact of pathologic complete response on prognosis after neoadjuvant chemotherapy in various intrinsic breast cancer subtypes. Journal of clinical oncology.

[22] Kocsis L, Svebis M, Boross G, Sinko M, Maraz R, Rajtar M, Cserni G (2004). Use and limitations of a nomogram predicting the likelihood of non-sentinel node involvement after a positive sentinel node biopsy in breast cancer patients. The American surgeon.

[23] Chen JY, Chen JJ, Yang BL, Liu ZB, Huang XY, Liu GY, Han QX, Yang WT, Shen ZZ, Shao ZM, Wu J (2012). Predicting sentinel lymph node metastasis in a Chinese breast cancer population: assessment of an existing nomogram and a new predictive nomogram. Breast cancer research and treatment.

[24] Babyak MA (2004). What you see may not be what you get: a brief, nontechnical introduction to overfitting in regression-type models. Psychosomatic medicine.

